# Efficient and Stable Antimony Selenoiodide Solar Cells

**DOI:** 10.1002/advs.202003172

**Published:** 2021-02-09

**Authors:** Riming Nie, Manman Hu, Andi Muhammad Risqi, Zhongping Li, Sang Il Seok

**Affiliations:** ^1^ School of Energy and Chemical Engineering Ulsan National Institute of Science and Technology (UNIST) 50 UNIST‐gil, Eonyang‐eup, Ulju‐gun Ulsan 44919 Republic of Korea

**Keywords:** chalcohalides, lead‐free perovskite materials, SbSeI, solar cells

## Abstract

Although antimony selenoiodide (SbSeI) exhibits a suitable bandgap as well as interesting physicochemical properties, it has not been applied to solar cells. Here the fabrication of SbSeI solar cells is reported for the first time using multiple spin‐coating cycles of SbI_3_ solutions on Sb_2_Se_3_ thin layer, which is formed by thermal decomposition after depositing a single‐source precursor solution. The performance exhibits a short‐circuit current density of 14.8 mA cm^−2^, an open‐circuit voltage of 473.0 mV, and a fill factor of 58.7%, yielding a power conversion efficiency (PCE) of 4.1% under standard air mass 1.5 global (AM 1.5 G, 100 mW cm^−2^). The cells retain ≈90.0% of the initial PCE even after illuminating under AM 1.5G (100 mW cm^−2^) for 2321 min. Here, a new approach is provided for combining selenide and iodide as anions, to fabricate highly efficient, highly stable, green, and low‐cost solar cells.

## Graphical abstract

The fabrication of SbSeI solar cells using multiple spin‐coating cycles of SbI_3_ solutions on Sb_2_Se_3_ thin layer (deposited by using a single‐source precursor solution) is reported. The champion cell exhibits a power conversion efficiency of 4.10% under standard air mass 1.5 global (100 mW cm^−2^). These cells show good humidity, photo‐, and thermal stabilities.

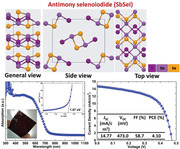

Small effective mass, large dielectric constant, high band dispersion level, and valence band maximum with antibonding states are desirable properties for highly efficient and defect‐tolerant light harvesters.^[^ ^1^ ^]^ Most of the aforementioned properties exist in materials containing metal cations with ns^2^ valence electron configuration,^[^ ^2^ ^]^ owing to their high bandwidth conduction band and high Born effective charge derived from large spin–orbit effects as well as their soft Polaris ability. Popular light harvesters including halides or chalcogenides of Pb^2+^,^[^ ^3^ ^]^ Sn^2+^,^[^ ^4^ ^]^ Ge^2+^,^[^ ^5^ ^]^ Sb^3+^,^[^ ^6^ ^]^ and Bi^3+[^ ^7^ ^]^ contain metal cations with the ns^2^ valence electron configuration. However, most of them are affected by one or several issues, such as low efficiency, low stability, toxicity, and high cost. Hence, efficient, stable, green, and low‐cost light‐harvesters must be developed.

As important material exhibiting the ns^2^ electronic configuration, metal chalcohalides have received extensive attention owing to their interesting physical and chemical properties.^[^ ^8^ ^]^ Because of the distinct bonding preferences of chalcogenide and halide atoms, to form stable sites in compounds, the competition among atoms might yield unique structures and properties.^[^ ^9^ ^]^ Additionally, a wide bandgap range can be obtained in these materials because halide and chalcogenide coexist as anions; hence, they can be used in various applications, such as solar cells, radiation detection and transparent electronic devices.^[^ ^10^ ^]^ As an important member of metal chalcohalides, SbSI has been applied to solar cells as a light harvester. SbSI solar cells with TiO_2_ and poly [2,6‐(4,4‐bis(2‐ethylhexyl)‐4H‐cyclopenta[2,1‐b;3,4‐b′]dithiophene)‐alt‐4,7‐(2,1,3‐benzothiadiazole)] (PCPDTBT) as electron transporting layer (ETL) and hole transporting layer (HTL), respectively, exhibit a relatively poor power conversion efficiency (PCE) of 3.05%.^[^ ^11^ ^]^ The unsatisfactory PCE might be due to the wide bandgap (≈2.1 eV) of SbSI. Compared with SbSI, SbSeI exhibits a much narrower bandgap (1.67 eV),^[^ ^12^ ^]^ which is more suitable for light harvesting. Furthermore, compared with the difference in ionic radii between S^2−^ (184 pm)^[^ ^13^ ^]^ and I^−^ (220 pm),^[^ ^13^ ^]^ the better match in ionic radii between Se^2−^ (198 pm)^[^ ^13^ ^]^ and I^−^ (220 pm) might produce superior and more stable crystal phases.

Furthermore, SbSeI is a representative A(V)B(VI)C(VII) type compound. Similar to SbSI, SbSeI is ferroelectric and a good photoconductor.^[^ ^14^ ^]^ SbSeI has attracted significant attention owing to its important optical and semiconducting properties, which have been applied in X‐ray and *γ*‐ray detections as well as optoelectronics.^[^ ^15^ ^]^ Based on relativistic quasi‐particle self‐consistent GW theory, Butler et al. reported that SbSeI exhibited a multivalley electronic structure, where several electron and hole basins occurred near the band extrema, which might cause nonconventional photophysical behaviors. The ionization potential of SbSeI was predicted to be 5.3 eV based on first‐principles calculations. Appropriate contact materials should be selected to optimize performance.^[^ ^16^ ^]^ Brandt et al. reported that the lifetime of SbSeI exceeded 1 ns, which is the threshold to yield promising early‐stage photovoltaic device performance.^[^ ^17^ ^]^ Although extensive studies have been performed regarding SbSeI, experimental studies applying SbSeI in solar cells are nonexistent.

In this study, we deposited SbSeI on the mesoporous (mp)‐TiO_2_ through multiple spin‐coating cycles of SbI_3_ solutions on Sb_2_Se_3_, which was formed by thermal decomposition after depositing a single‐source precursor (Se‐SSP). Here, [(SbL_2_Cl_2_)Cl]_2_·(CH_3_)_2_CO], where L is *N*,*N*‐dimethyl selenourea, as Se‐SSP was synthesized according to our previous work.^[^ ^18^ ^]^ SbSeI was characterized by X‐ray powder diffraction (XRD), ultraviolet–visible (UV–vis) absorption spectroscopy, X‐ray photoelectron spectroscopy (XPS), high‐resolution transmission electron microscopy (HR‐TEM), field emission scanning electron microscopy (FESEM) and ultraviolet photoelectron spectroscopy (UPS). The effects of deposition cycles including the spin‐coating and thermal decomposition of SbSeI on the device performance were investigated by measuring the electrochemical impedance spectroscopy (EIS) and temperature‐dependent current–voltage curves. The cells with TiO_2_ and PCPDTBT as the ETL and the HTL, respectively, exhibited a PCE of 4.10%. Furthermore, the cells showed good stabilities under ambient conditions (≈80% relative humidity (RH)), at 85 °C in air (<30% average RH), and under standard air mass 1.5 global (AM 1.5 G) conditions (100 mW cm^−2^) without a UV light filter.

**Figure** **1**a shows the crystal structure of SbSeI from general, side, and top views. Figure 1b shows the XRD pattern of the glass/mp‐TiO_2_/SbSeI in the range of 10°–60°; it is well‐indexed to the SbSeI orthorhombic phase with a Pnma (62) group (JCPDS No. 76‐1354). To observe the main peaks, the XRD patterns in the range of 18°–22° and 28°–32.5° are shown in Figure 1c. The crystal structure of SbSeI is very similar to that of Sb_2_Se_3_ (Figures S1 and S2, Supporting Information) except for lattice parameters. The UV–vis absorption spectrum of the glass/mp‐TiO_2_/SbSeI is shown in Figure 1d, and the insets show the corresponding Tauc plot and photograph. SbSeI had a bandgap of 1.67 eV, as calculated from the transmission spectrum (Figure S3, Supporting Information). The bandgap of SbSeI matched well with its dark brown color and the reference value.^[^ ^12^ ^]^ As shown in Figure S4 (Supporting Information), the band gap of SbSeI remains unchanged even when excessive SbI_3_ is applied. Figure 1e,f shows the HR‐TEM images of SbSeI located at mp‐TiO_2_, revealing the crystal structure of SbSeI. The d‐spacing of the crystal lattice of 0.303 nm was assigned to the (112) plane of the orthorhombic phase of the SbSeI crystal. The uniform distribution of SbSeI on mp‐TiO_2_ was confirmed by scanning transmission electron microscopy (STEM) and energy‐dispersive X‐ray (EDX) elemental mapping (Figure 1g). The EDX data showed that the Sb:Se:I ratio was 37.0:36.9:26.1 (Figure S5, Supporting Information), indicating that I was slightly less than the theoretical 1:1:1 ratio. This may be related to the small amount of Sb_2_Se_3_ remaining in SbSeI. Therefore, since a small amount of Sb_2_Se_3_ is expected to coexist under the current experimental conditions, further research is needed to prepare high‐purity SbSeI. Figures S6 and S7 in the Supporting Information show the survey and high‐resolution XPS spectra of fluorine doped tin oxide (FTO)/mp‐TiO_2_/SbSeI. Additionally, peaks corresponding to Sb, Se, and I were observed, which matched well with the reference spectra.^[^ ^19^ ^]^ The Ti 2p peaks were acquired from mp‐TiO_2_, whereas the C 1s peaks were owing to adventitious carbon contamination.^[^ ^20^ ^]^ Figure S8 in the Supporting Information shows the process for depositing SbSeI.

**Figure 1:**
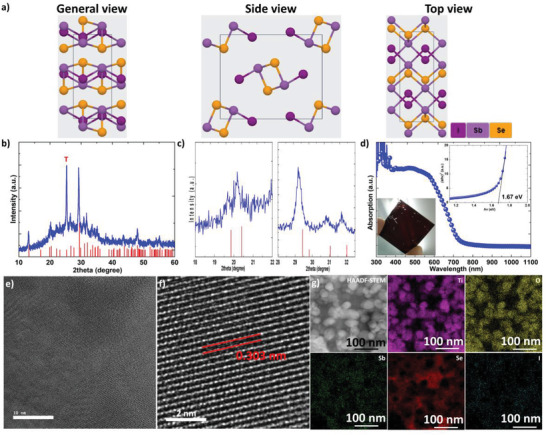
Confirmation of SbSeI. a) Schematic diagrams of crystal structure of SbSeI in general, side, and top views. b,c) XRD patterns at ranges of 10°–60°, 18°–22°, and 28°–32.5°, and d) UV–vis absorption spectrum of glass/mp‐TiO_2_/SbSeI. Standard SbSeI (JCPDS No. 76‐1354) structure file is shown as the red column in (c). Main peak of TiO_2_ at 25.3° is marked as “T” in (c). Insets in (d) show the corresponding Tauc plot and a photograph of the glass/mp‐TiO_2_/SbSeI. e) Low magnification and f) high magnification HR‐TEM of SbSeI on mp‐TiO_2_. g) HAADF–STEM, and corresponding EDX mapping images of SbSeI on mp‐TiO_2_.

The surface FESEM image of the glass/mp‐TiO_2_/SbSeI is shown in **Figure** **2**a. SbSeI was located on mp‐TiO_2_. Some SbSeI needles were observed, as SbSeI tends to grow in the form of long needles along the *c*‐axis of the material.^[^ ^21^ ^]^ Figure 2b shows the cross‐sectional FESEM image of the SbSeI solar cells, where SbSeI was employed as a light‐harvesting material. Additionally, PCPDTBT (10 mg in 1 mL of dichlorobenzene) and poly(3,4‐ethylenedioxythiophene) doped with poly(4‐styrenesulfonate) (PEDOT:PSS) were used as the hole‐transporting material (HTM) and HTL, respectively.^[^ ^22^ ^]^ Furthermore, an FTO layer, a TiO_2_ blocking layer, an mp‐TiO_2_/SbSeI/HTM(L) layer, and an Au layer were observed. The uniform morphology of the mp‐TiO_2_/SbSeI/HTM(L) indicated the uniform distribution of SbSeI in mp‐TiO_2_ and the efficient infiltration of HTM(L) into the pores of mp‐TiO_2_. To further confirm the uniform distribution of SbSeI in mp‐TiO_2_, the line EDX data (Figure 2c) scanned from the top to bottom in the mp‐TiO_2_/SbSeI/HTM(L) layer and the EDX mapping analysis (Figure 2d) acquired from the yellow rectangle in Figure 2b were also carried out; consequently it was observed that Sb, Se and I were uniformly distributed. In addition, the uniform distribution was also confirmed in the vertical direction of the device by EDX measurements at the bottom, middle and top of the glass/mp‐TiO_2_/SbSeI (Figure S9, Supporting Information). Because UPS is more surface sensitive than XPS, XPS and UPS were used to measure the energy levels. In particular, XPS was used to detect the highest occupied molecular orbital (HOMO) regions to avoid the effect of surface contamination. Figure 2e shows magnified plots of the secondary electron cut‐off regions of the He I UPS spectrum and the XPS valance level spectrum of SbSeI. The Fermi level (*E*_F_) of SbSeI was 4.65 eV, with the valence band maximum (VBM) located at 1.04 eV below the *E*_F_ (This value is very close to that obtained from the HOMO region of He I UPS spectra (Figure S10, Supporting Information)). The *E*_F_ of SbSeI was measured twice; it was discovered that their values were similar, indicating that the *E*_F_ value was reliable (Figure S11, Supporting Information). Combining these results and the bandgap value (Figure 1d), the energy levels of SbSeI were obtained (Figure 2f). The conduction band minimum (CBM) of SbSeI was 4.02 eV, and the VBM was 5.69 eV. The gap between CBM and *E*_F_ is narrower than that between VBM and *E*_F_, indicating that the conductivity type of SbSeI prepared in this work is n‐type, which agrees well with previously reported results.^[^ ^15a^ ^]^ Therefore, the energy level of SbSeI is expected to be effective charge transfer to CBM of TiO_2_ and HOMO of HTM.

**Figure 2:**
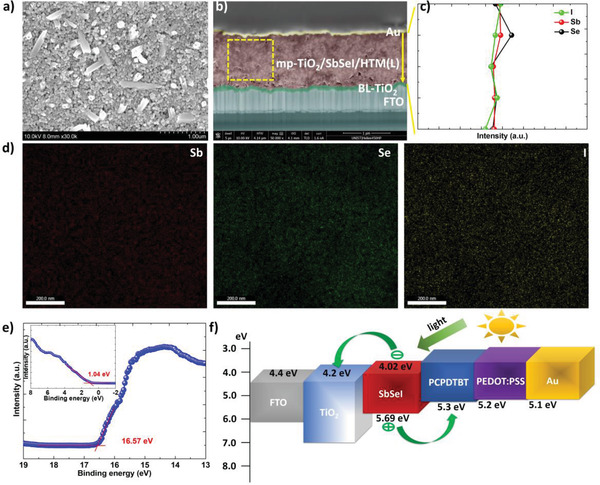
Distribution and energy level. a) Surface SEM image of glass/mp‐TiO_2_/SbSeI. b) Cross‐sectional SEM image of FTO/BL/mp‐TiO_2_/SbSeI/HTM(L)/Au solar cell. c) EDX line data scanned from the top to bottom in mp‐TiO_2_/SbSeI/HTM(L) layer in (b). d) EDX mapping data acquired from yellow rectangle in (b). e) Secondary electron cut‐off region of He I UPS spectra and XPS valence level spectrum for FTO/mp‐TiO_2_/SbSeI. f) Energy levels of the functional materials employed in FTO/BL/mp‐TiO_2_/SbSeI/HTL/Au solar cells.

The effect of SbSeI loading amount on the device performance was investigated. The performance of the Sb_2_Se_3_ solar cell was also shown in Figure S12, Supporting Information. **Figure** **3**a shows the current density–voltage (*J*–*V*) curves of the SbSeI solar cells, where SbSeI was prepared using 8, 10, and 12 multicycles of spin‐coating and thermal decomposition. The device performances are summarized in **Table** **1**. In the case of 8 cycles, the short‐circuit current density (*J*_SC_), open‐circuit voltage (*V*_OC_), and fill factor (FF) were 11.7 mA cm^−2^, 443.8 mV and 54.1%, respectively, resulting in a PCE of 2.81%. As the number of cycles increased to 10, the PCE increased to 3.77%, accompanied by simultaneous increments in the *J*_SC_, *V*_OC_, and FF. When the cycle further increased to 12, the PCE decreased to 1.23%, with significant decrements in the *J*_SC_, *V*_OC_, and FF. To understand the reason contributing to the changed device performances, we first measured the incident photon‐to‐electron conversion efficiency (IPCE) of these cells, which is closely related to the *J*_SC_. As shown in Figure 3b, as the number of cycles increased from 8 to 10, the IPCE spectrum were shifted to upper values in the entire range. When it further increased to 12, the IPCE spectrum decreased. The change in the IPCE spectra matched well with the *J*_SC_ trend. Light‐harvesting efficiency (LHE), electron injection yield (EIY), and charge collection efficiency (CCE) are three key factors that determine the IPCE spectra. Furthermore, EIY and CCE can be combined as the charge‐transfer yield (CTY). Figure 3c shows the absorption spectra of the glass/mp‐TiO_2_/SbSeI prepared through 8, 10, and 12 spin‐coating cycles and thermal decomposition. As the number of cycles increased from 8 to 10, the improved *J*_SC_ could be attributed to the enhanced LHE, which was confirmed by the increased absorption. However, further increasing the number of cycles to 12 did not increase the absorption. Hence, the reduction in the PCE of the cells fabricated through 12 cycles was attributable to the reduced CTY. To investigate the CTY, we obtained the electrochemical impedance spectra of those cells. Figure 3d shows the Nyquist plots of the SbSeI solar cells prepared through 10 and 12 spin‐coating cycles and thermal decomposition; the cells were measured at a bias of 0.2 V in the frequency range from 100 kHz to 0.1 Hz in the dark. The Nyquist plots were fitted using the equivalent circuit shown in the inset. The high‐frequency section in the Nyquist curves represents the series resistance (*R*_s_), whereas the low‐frequency section represents the recombination resistance (*R*_REC_).^[^ ^23^ ^]^ The fitting parameters of the Nyquist curves are shown in Table S1 in the Supporting Information. The cell prepared through 10 cycles yielded a greater *R*_REC_ than that through 12 cycles, indicating a more efficient interface charge transfer in the cell prepared through 10 cycles. To understand the reason contributing to the different CTYs between the cells prepared through 10 and 12 cycles, we analyzed their trap states using temperature‐dependent current–voltage curves. Figure S13 in the Supporting information shows the current–voltage curves at various temperatures in the presence and absence of light irradiation. The average activation energy of the trapped electrons can be calculated using the Richardson–Dushman equation as follows^[^ ^24^ ^]^

(1) $$J\propto e^{-\Delta E/kT}$$

where Δ*E*, *k*, and *T* are the electron activation energy, Boltzmann constant, and absolute temperature, respectively. Because the current at 0 V can be easily affected by noise, we used the current at −0.5 V bias to calculate Δ*E*. Figure 3e,f shows the dependence of the dark current and photocurrent at −0.5 V bias for the cells prepared through 10 and 12 cycles on temperature, respectively. The slopes of the fitted lines were used to calculate the activation energy. The activation energies in the dark and under illumination for the cells prepared through 10 cycles were 0.340 and 0.0167 eV, respectively, and the corresponding values for the cells prepared through 12 cycles were 0.412 and 0.0968 eV, respectively. Compared with the cells prepared through 10 cycles, those prepared through 12 cycles showed deeper level traps, which might have contributed to their lower CTYs. To illustrate the reason contributing to the different device performances of these cells, we sketched several pictures (Figure 3g). In the case of 8 cycles, the poor PCE values were obtained due to insufficient absorption. The cells prepared through 10 cycles exhibited the highest PCE owing to sufficient absorption and efficient charge transfer. Although the cells prepared through 12 cycles indicated sufficient absorption, their charge transfer was inefficient, which might be due to the inefficient infiltration of HTM induced by excess light absorbers, according to our previous study.^[^ ^25^ ^]^

**Figure 3:**
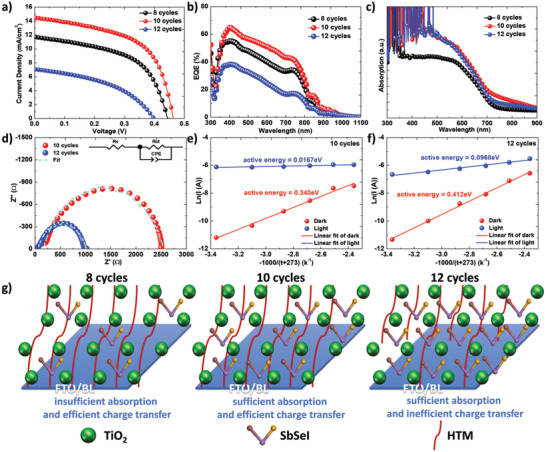
Effect of SbSeI loading amount on the device performance. a) *J*–*V* curves under standard illumination conditions (100 mW cm^−2^) of AM 1.5 G and b) IPCE spectra of SbSeI solar cells fabricated through 8, 10, and 12 spin‐coating cycles and thermal decomposition. c) UV–vis absorption spectra of the glass/mp‐TiO_2_/SbSeI prepared by 8, 10, and 12 cycles of spin‐coating and thermal decomposition processes. d) Nyquist plots under dark condition. e,f) Dependence of dark current and photo current on temperature of SbSeI devices fabricated through 10 and 12 spin‐coating cycles and thermal decomposition. The inset in (d) shows an equivalent circuit used to fit impedance curves. g) Schematic diagrams of light‐harvesting and charge‐transfer processes in SbSeI solar cells fabricated through 8, 10, and 12 spin‐coating cycles and thermal decomposition.

**Table 1:** Performance of SbSeI solar cells fabricated through 8, 10, and 12 spin‐coating cycles and thermal decomposition. Here *J*_SC_, *V*_OC_, FF, and PCE denote short‐circuit current density (*J*sc), open‐circuit voltage(*V*_oc_), fill factor (FF), and power conversion efficiency (PCE), respectively

Deposition multicycle^a)^	*J*_SC_ [mA cm^−2^]	*V*_OC_ [mV]	FF [%]	PCE [%]
8 cycles	11.7	443.8	54.1	2.81
10 cycles	14.5	463.2	56.1	3.77
12 cycles	7.1	398.4	43.5	1.23

^a)^ The deposition cycle for preparing SbSeI, including spin‐coating and thermal decomposition.

The thickness of mp‐TiO_2_, and the concentration of the Se‐SSP solution were optimized (Figure S14 and S15, Supporting Information). **Figure** **4**a depicts the *J*–*V* curve of the best‐performing SbSeI solar cell, which exhibited a PCE of 4.10%, with a *J*_SC_ of 14.77 mA cm^−2^, a *V*_OC_ of 473.0 mV, and an FF of 58.7%. This PCE is the highest value among the solar cells that were first demonstrated by applying new light absorbers. (Table S2, Supporting Information). The corresponding IPCE spectrum is shown in Figure 4b. The IPCE spectrum beyond the SbSeI absorption could be attributed to the additional absorption from PCPDTBT.^[^ ^22^ ^]^ Integrating the overlap of the standard AM 1.5 G solar photon flux with the IPCE spectrum yielded a current density of 14.66 mA cm^−2^, which was similar to the value measured from the *J*–*V* curve. Figure 4c shows the stabilized power output of the SbSeI solar cell, which exhibited a stabilized current density of 10.51 mA cm^−2^ and a stabilized PCE of 3.95% achieved by maintaining the bias voltage at the maximum power point (0.376 V). Here, a cooling fan was used to minimize the temperature rise due to continuous light irradiation. The PCE obtained from the steady‐state measurement was consistent with the *J*–*V* measurements, indicating that the PCEs of the SbSeI solar cells are reliable. Figure 4d shows the PCE distribution of the SbSeI solar cells; a normal distribution was observed.

**Figure 4:**
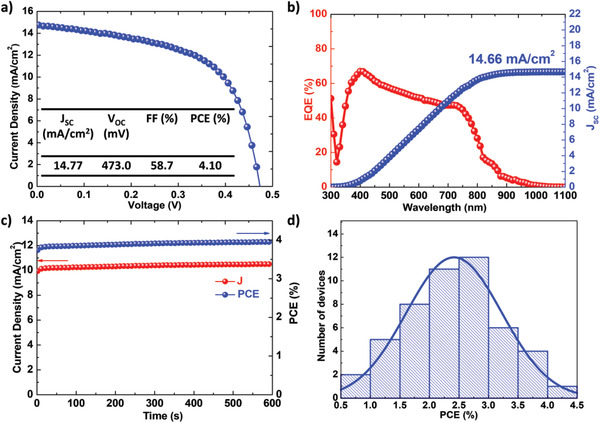
The best‐performing cell. a) *J*–*V* characteristic under standard illumination conditions (AM 1.5 G, 100 mW cm^−2^) of SbSeI solar cell and b) corresponding external quantum efficiency (EQE) spectrum. The device performance parameters are listed in the inset. c) Stabilized power output of SbSeI solar cell by maintaining the voltage at maximum power point (0.376 V). d) Histogram of device efficiencies from 49 individual cells.

To check the humidity stability, the cells without encapsulation were stored under ambient conditions (≈80% relative humidity) at room temperature in the dark. As shown in **Figure** **5**a,b, the SbSeI solar cells retained 89.3% of the initial PCE after storage for 360 h, which was primarily attributable to the decreased *V*_OC_. Figure 5c,d shows the thermal stability of the unencapsulated cells stored at 85 °C in air (<30% average RH). The cells retained 89.6% of the initial PCE after being stored for 672 h, accompanied by significantly reduced *J*_SC_, and almost unchanged *V*_OC_ and FF. Additionally, the unencapsulated devices were illuminated under standard AM 1.5 G conditions (100 mW cm^−2^) without a UV light filter to test their photostability. After illuminating for 2321 min, the cells retained 90.0% of the initial PCE. These results show that the SbSeI solar cells exhibited good stabilities, regardless of humidity, temperature, and light.

**Figure 5:**
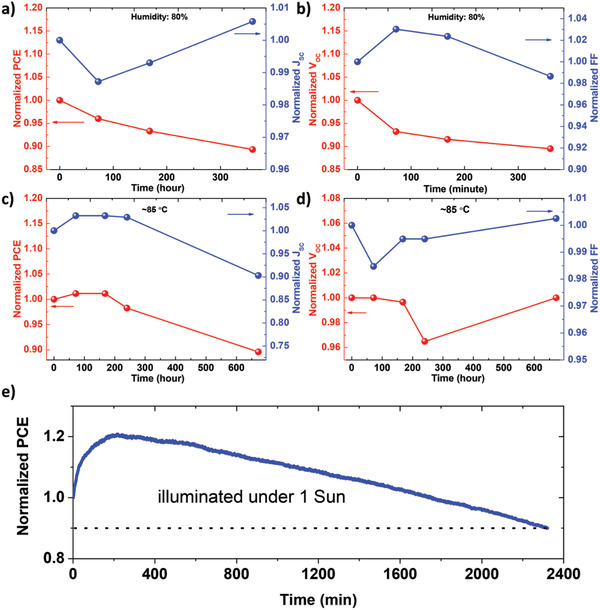
Long‐term stability. Changes in normalized device performance of unencapsulated SbSeI solar cells as storage time increases: a,b) in ambient atmosphere (≈80% humidity) and in the dark at room temperature, c,d) measured at 85 °C in air (<30% relative humidity), and e) under standard AM 1.5G illumination using a Xenon lamp including UV light.

In summary, SbSeI solar cells were fabricated for the first time using multiple spin‐coating cycles of a SbI_3_ solution on Sb_2_Se_3_ and thermal annealing. Subsequently, Sb_2_Se_3_ was deposited using a Se‐SSP solution. SbSeI is of the orthorhombic phase with a Pnma (62) group and has a bandgap of 1.67 eV. The SbSeI cells fabricated by 8 and 12 spin‐coating cycles and thermal decomposition exhibited poor PCEs due to insufficient absorption and inefficient charge transfer, respectively. The inefficient charge transfer in the cells fabricated through 12 cycles was due to deeper level trap states. Sufficient absorption and efficient charge transfer were achieved in the cells fabricated through 10 cycles, and the champion cell exhibited a PCE of 4.10%. Furthermore, these cells demonstrated excellent humidity, thermal, and photo stabilities. This study provides an insight into using metal chalcohalides for solar cells and intermediate products to synthesize lead‐free perovskite materials.

## Experimental Section

##### Preparation of FTO/TiO_2_‐BL/mp‐TiO_2_

To deposit a 100 nm thick TiO_2_ blocking layer (TiO_2_‐BL), pray pyrolysis of 20 × 10^−3^ m titanium diisopropoxide bis(acetylacetonate) (Aldrich) solution was performed on an FTO glass substrate at 450 °C. After cooling, an mp‐TiO_2_ layer with a thickness in the range of 150–1800 nm was prepared by a screen‐printed TiO_2_ paste comprising TiO_2_ nanoparticles (average diameter 50 nm, anatase). Subsequently, the substrates were annealed at 500 °C for 1 h in air to crystallize TiO_2_ and remove organic materials.

##### Preparation of FTO/TiO_2_‐BL/mp‐TiO_2_/SbSeI

An Se‐SSP was prepared according to the method detailed in the previous study.^[^ ^18^ ^]^ The Se‐SSP solution in DMF (0.05–0.2 mol L^−1^) was spin‐coated on FTO/BL/mp‐TiO_2_ substrates at 1000–2000 rpm for 60 s. Subsequently, the substrates were thermally decomposed in Ar gas at 150 °C for 2 min. The spin‐coating cycle and thermal decomposition were repeated to obtain the appropriate amount of Sb_2_Se_3_. Subsequently, the samples were annealed in Ar at 300 °C for 10 min to crystallize Sb_2_Se_3_. To deposit SbSeI, the SbI_3_ solution (0.05–0.2 mol L^−1^) was spin‐coated on FTO/BL/mp‐TiO_2_/Sb_2_Se_3_ at 1000–2000 rpm for 60 s; subsequently, the samples were annealed in Ar gas at 150 °C for 2 min. The spin‐coating cycle and thermal decomposition for the SbI_3_ solution were identical to those for Se‐SSP. The concentration of the SbI_3_ solution was identical to that of the Se‐SSP solution. Finally, the samples were washed in DMF to remove the remaining SbI_3_.

##### Device Fabrication

To deposit the hole‐transporting material, a solution containing 10 mg of PCPDTBT (one material) and 10 mg of PC_71_BM (nano C 1,2‐dichlorobenzene) in 1 mL of 1,2‐dichlorobenzene was spin‐coated onto FTO/TiO_2_‐BL/mp‐TiO_2_/SbSeI at 2000 rpm for 60 s. Subsequently, the HTL was prepared by spin‐coating a poly(3,4‐ethylenedioxythiophene) doped with a poly(4‐styrenesulfonate) (PEDOT:PSS; Baytron AI 4083) solution at 2000 rpm for 60 s and then diluted threefold in MeOH. Finally, the anode was prepared by thermal depositing a 100 nm thick Au layer onto the samples as the anode. The active area of the device measured 16 mm^2^.

##### Material and Device Characterization

Crystal structure, absorption, microscopic structure, morphology, surface state, and energy level were characterized using a powder X‐ray diffractometer (D/MAX2500V/PC, Rigaku, Japan), UV–vis spectrophotometer (Jasco V‐780), high‐resolution transmission electron microscope (JEM‐2100F, JEOL, Japan), field‐emission scanning electron microscope (Hitachi High‐Technologies, S‐4800), and Thermo‐Fisher machine (ESCLAB 250XI) with either a monochromatic Al K*α* source (1486.6 eV) or an unfiltered He I (21.22 eV) gas discharge lamp. High angle annular dark field (HAADF) STEM images were obtained using an FEI Titan3 G2 60‐300 instrument equipped with a probe‐side spherical aberration corrector operating at an accelerating voltage of 200 kV. EDX elemental mapping was conducted using FEI's Super‐X EDS detection system. Elemental analysis was conducted using a Thermo Scientific Flash 2000 analyzer. The cross‐sectional image and corresponding EDX line and mapping data were acquired using a focused ion beam instrument (Helios NanoLab 450, FEI) and energy dispersive spectroscopy. The *J*–*V* curves and IPCE spectra were measured using a solar simulator (Newport, Oriel Class A, 91195A) with a source meter (Keithley 2400) at 100 mA cm^−2^ illumination AM 1.5 G and an internal quantum efficiency system (Oriel, IQE 200B), respectively. The electrochemical impedance spectra were measured using an AUTO LAB (AUT302N).

## Conflict of Interest

The authors declare no conflict of interest.

## Supporting information

Supporting Information
