# Supplementary material for: Efficient and Stable Antimony Selenoiodide Solar Cells
Source: Adv Sci (Weinh). 2021 Feb 9;8(8):2003172. doi: 10.1002/advs.202003172 (PMC8061406; doi:10.1002/advs.202003172)
Supplement: Supplementary file 1 — Supporting Information [file ADVS-8-2003172-s001.pdf]

WILEY-VCH

© 2020 Wiley-VCH GmbH

ADVANCED SCIENCE
Open Access

Supporting Information

for *Adv. Sci.*, DOI: 10.1002/advs.202003172

Efficient and Stable Antimony Selenoiodide Solar Cells

*Riming Nie, Manman Hu, Andi Muhammad Risqi, Zhongping Li, and Sang Il Seok**

Copyright © 2020 Wiley-VCH GmbH

Supporting Information

Efficient and Stable Antimony Selenoiodide Solar Cells

Riming Nie, Manman Hu, Andi Muhammad Risqi, Zhongping Li, and Sang Il Seok[*]

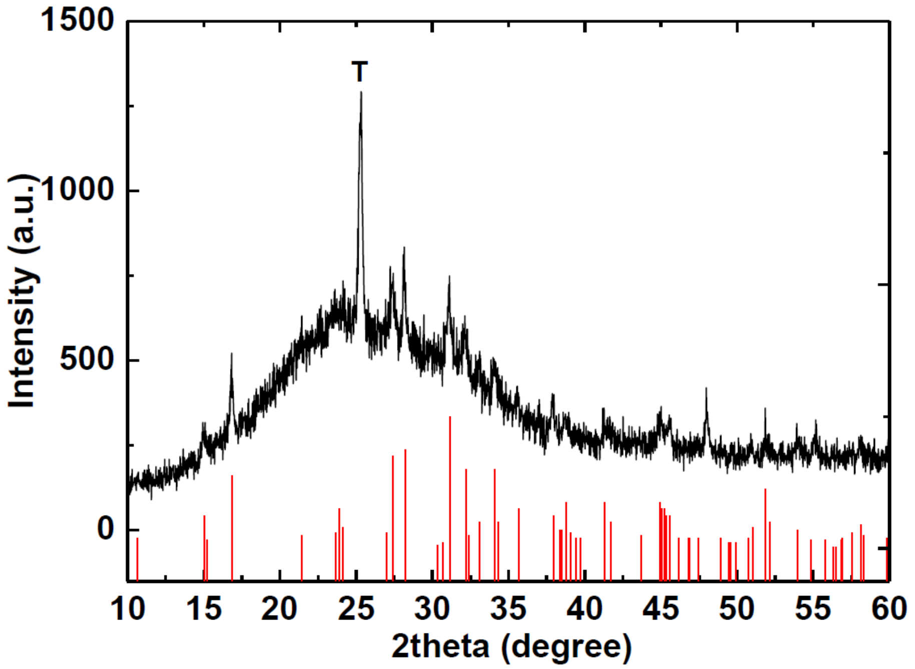

Figure S1. XRD patterns measured with the glass/mp-TiO_2/Sb_2Se_3. The reported Sb_2Se_3 structure (JCPDS No. 15-0861) was plotted as the red column. The main peak of TiO_2 at 25.3° was marked as "T".

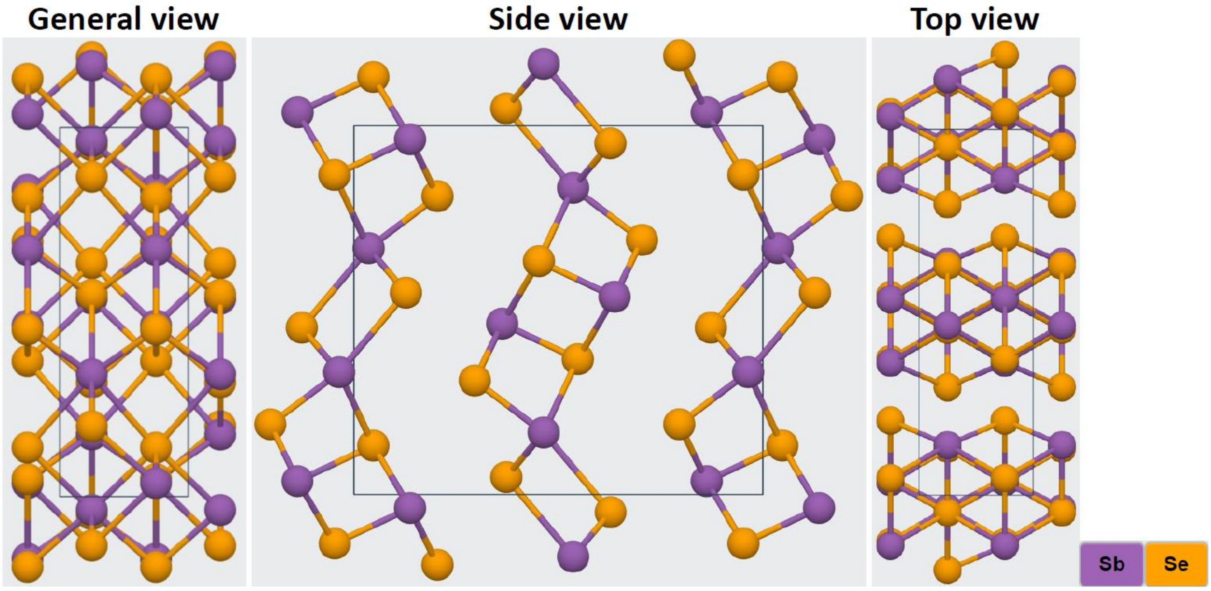

Figure S2. Schematic diagrams of Sb_2Se_3 crystal structure. SbSeI shows an orthorhombic crystal structure with a Pnma [62] group. The lattice parameters are as follows: a = 4.187 Å, b = 9.164 Å, c = 11.100 Å, α = 90.000°, β = 90.000° and γ = 90.000°. Sb_2Se_3 also shows an orthorhombic crystal structure with a Pnma [62] group. The lattice parameters are as follows: a = 4.030 Å, b = 11.541 Å, c = 12.843 Å, α = 90.000°, β = 90.000°, γ = 90.000°.

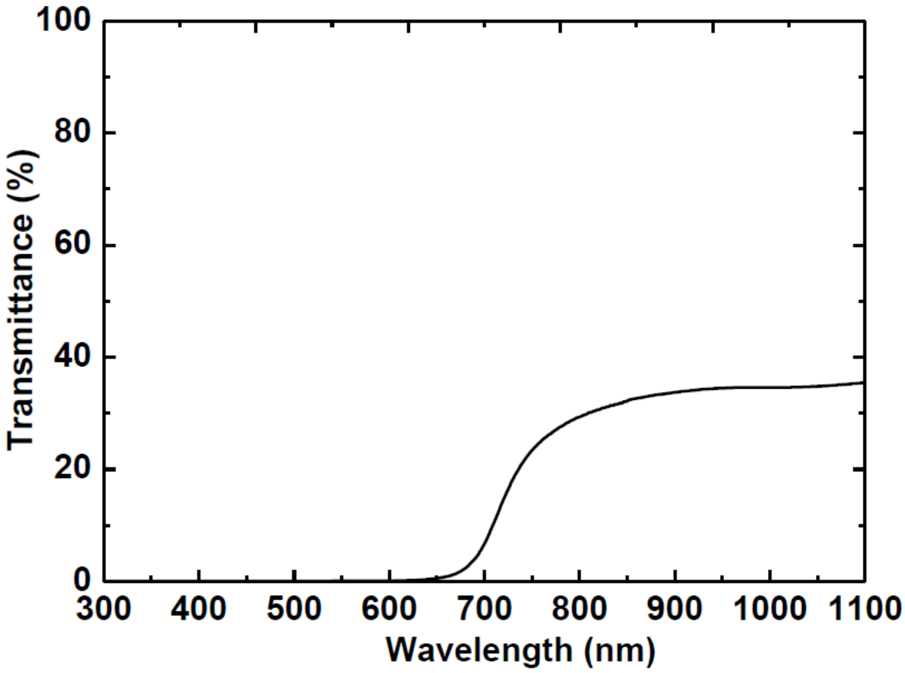

Figure S3. Transmission spectrum of the glass/mp-TiO_2/SbSeI.

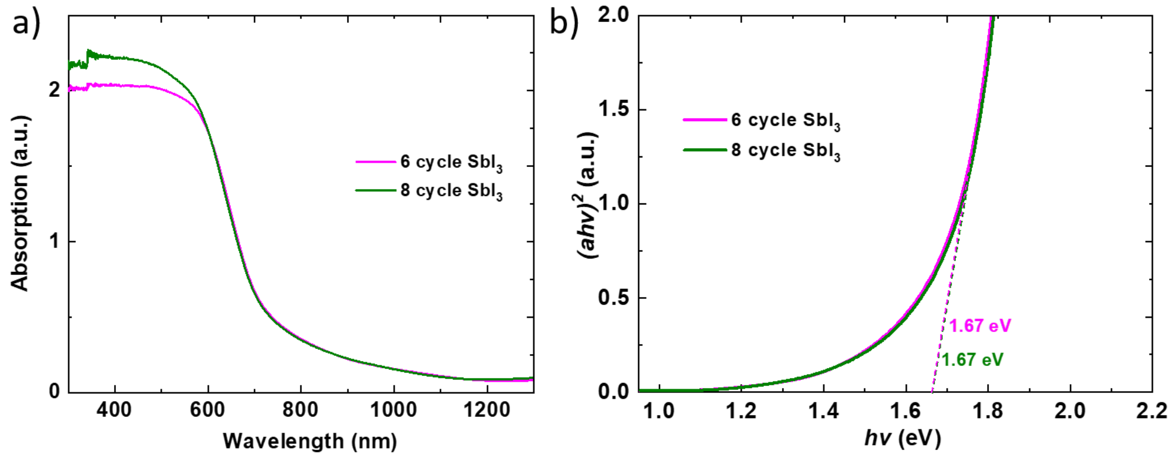

Figure S4. a) UV-vis absorption spectrum and b) the corresponding Tauc plot of the glass/mp-TiO_2/SbSeI prepared by 6 and 8 cycle of spin-coating SbI_3.

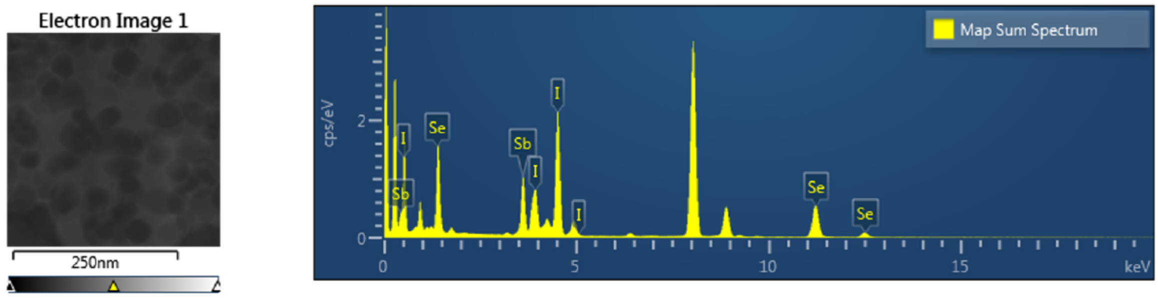

Element	Line Type	k Factor	k Factor type	Absorption Correction	Wt%	Wt% Sigma	Atomic %
Se	L series	1.707		1.00	27.24	0.19	37.01
Sb	L series	1.940		1.00	41.90	0.22	36.91
I	L series	1.969		1.00	30.86	0.22	26.08
Total:					100.00		100.00

Figure S5. EDX data acquired from the HRTEM attached energy dispersive spectroscopy.

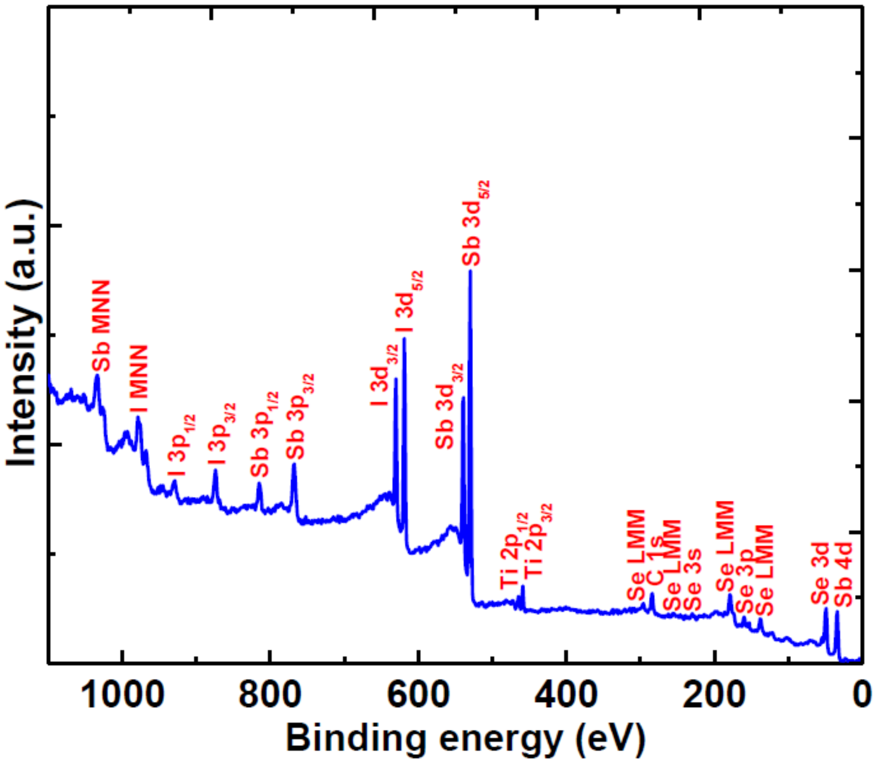

Figure S6. Survey XPS spectra of the FTO/mp-TiO_2/SbSeI.

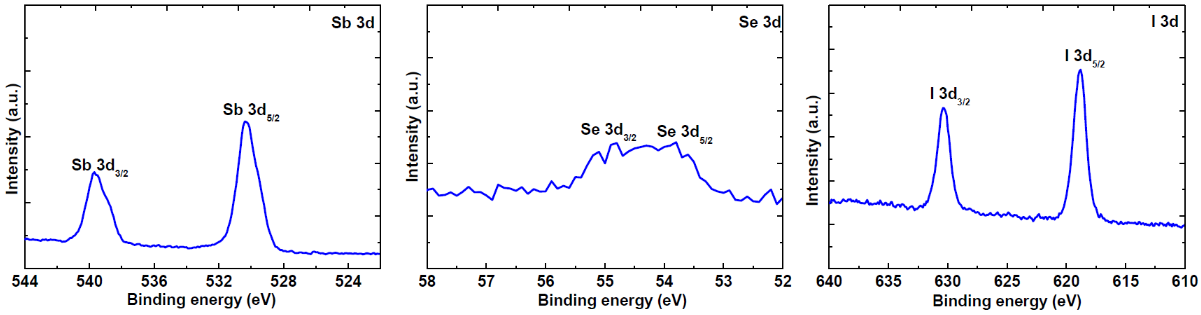

Figure S7. High resolution XPS spectra of the FTO/mp-TiO_2/SbSeI. a) Sb 3d, b) Se 3d, and c) I 3d.

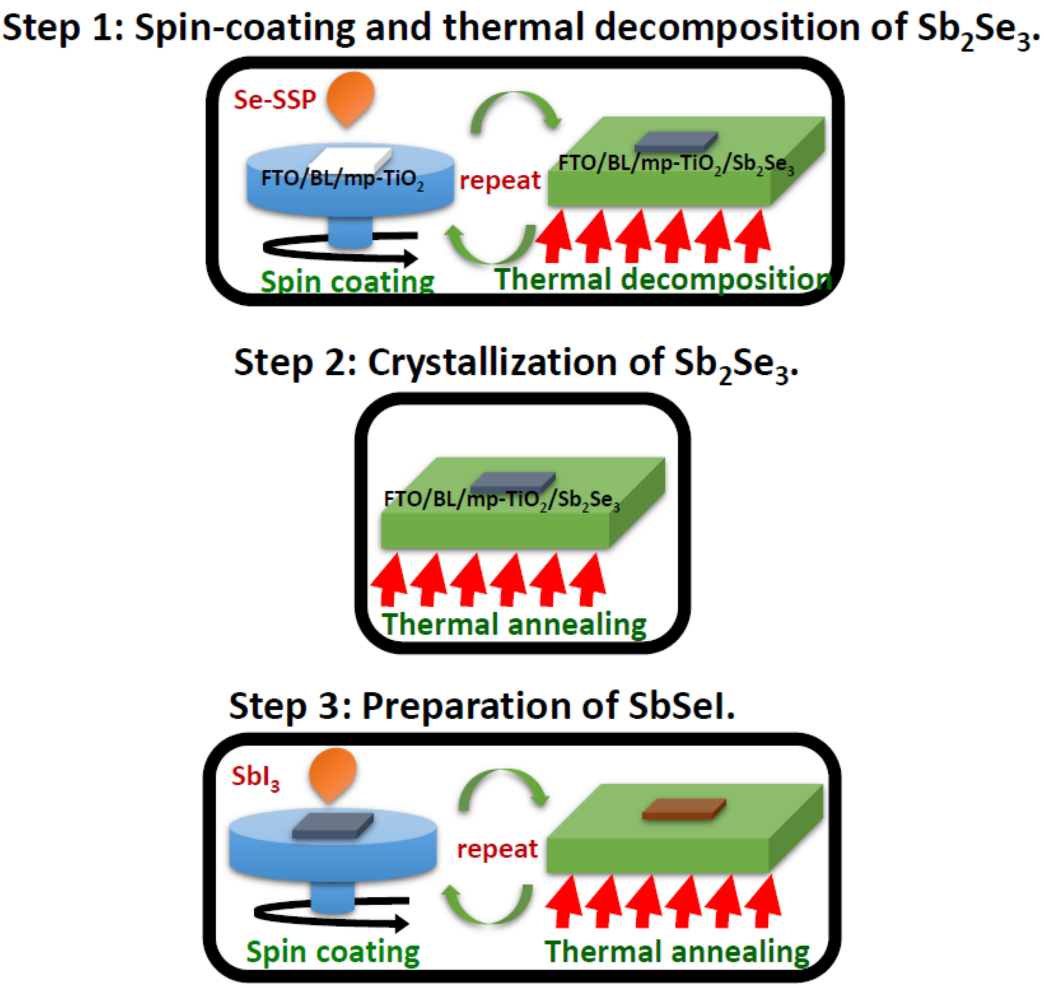

Figure S8. The process for depositing SbSeI.

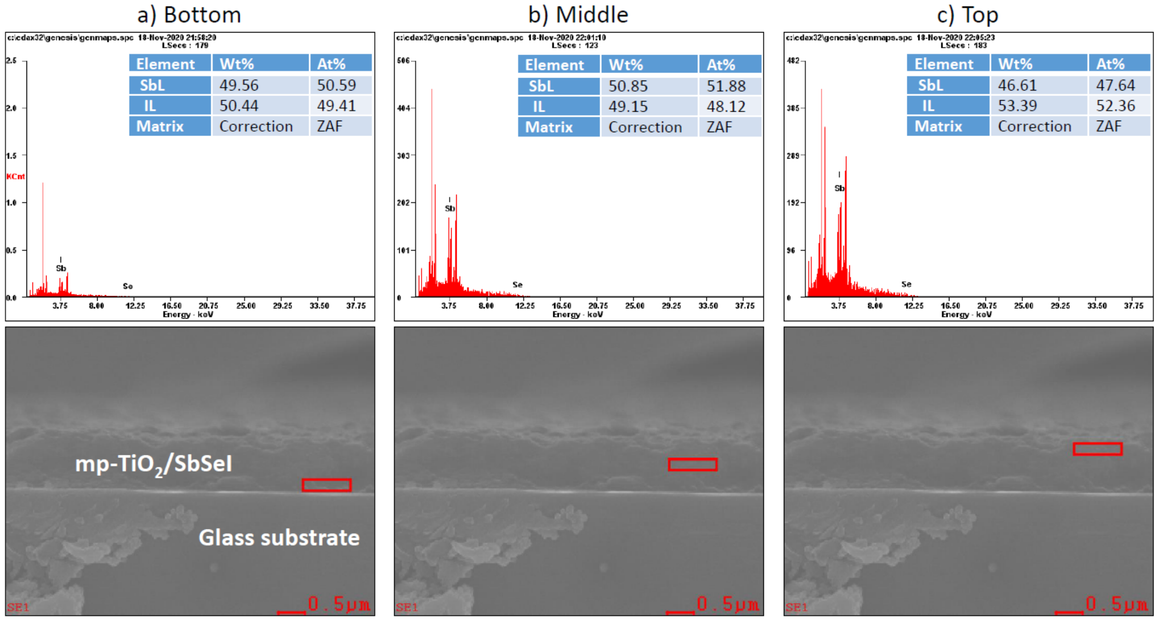

Figure S9. EDX data acquired at the a) bottom, b) middle and c) top of the glass/mp-TiO_2/SbSeI sample. It is not easy to detect Se. As a whole, at bottom, middle and top of mp-TiO_2/SbSeI, the ratio of Sb and I is close to 1:1, which matches well with the composition of SbSeI, indicating that SbSeI is uniformly distributed in mp-TiO_2. However, I exists in a slightly higher ratio than Sb in the top part, but the I ratio is deficient in the middle and bottom, because SbSeI is formed by diffusion by coating SbI_3 on the surface of the Sb_2Se_3 thin film.

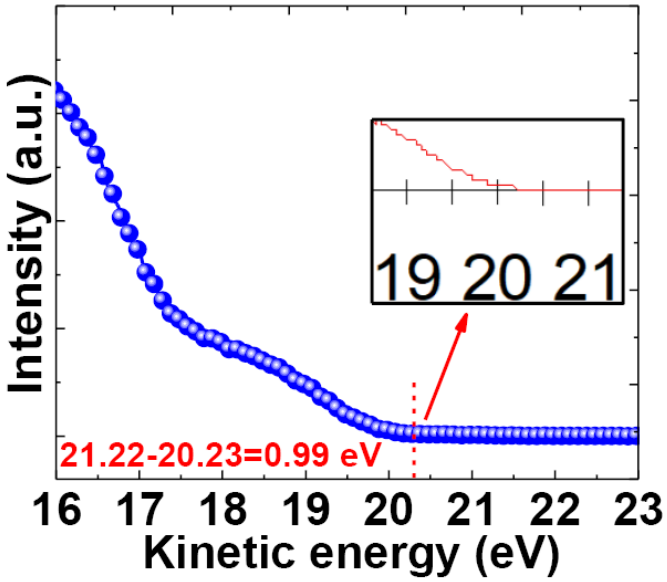

Figure S10. Highest occupied molecular orbital (HOMO) region of He I UPS spectra for FTO/mp-TiO_2/SbSeI.

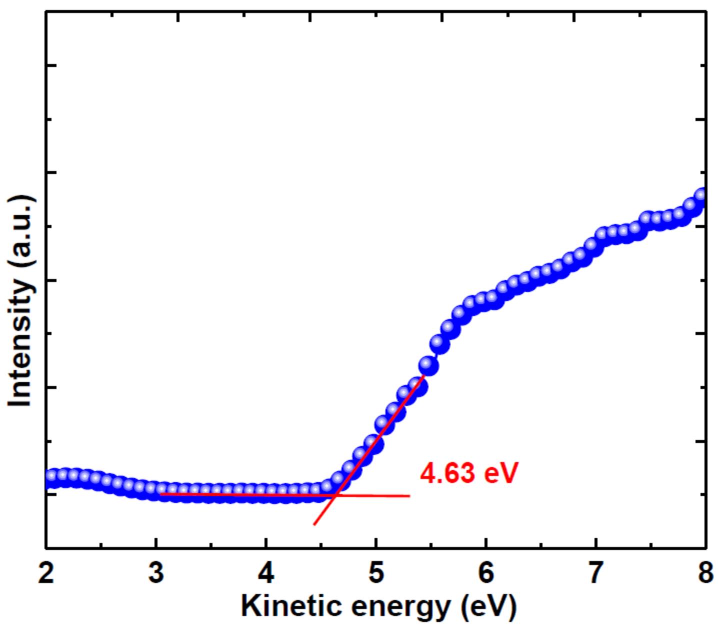

Figure S11. Secondary electron cut-off region of He I UPS spectra for FTO/mp-TiO_2/SbSeI acquired from second measurement.

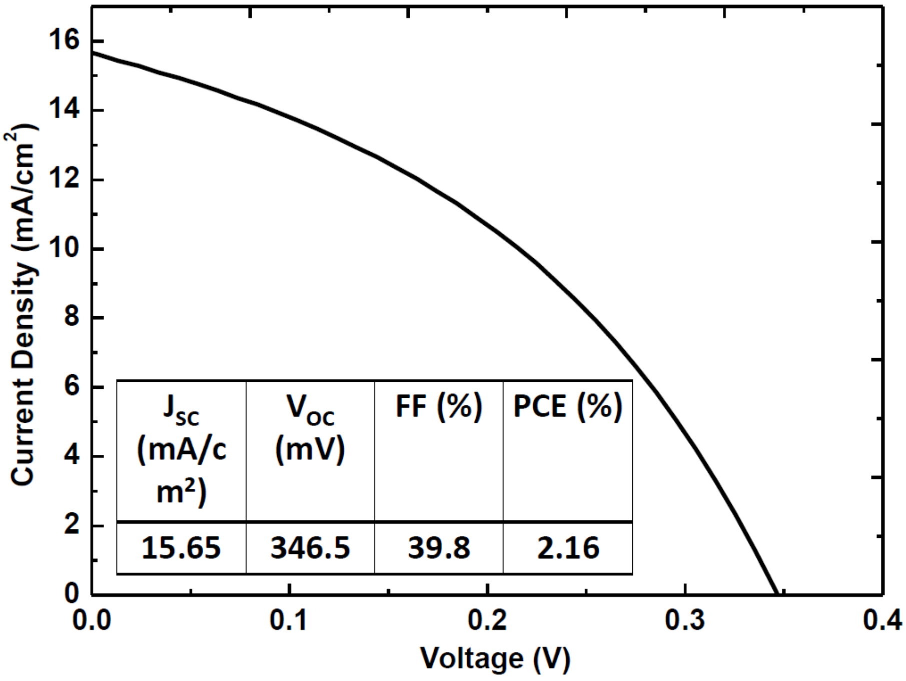

Figure S12. J-V curve under standard illumination conditions (AM 1.5 G, 100 mW cm^{-2}) of the Sb_2Se_3 solar cell without SbI_3 treatment.

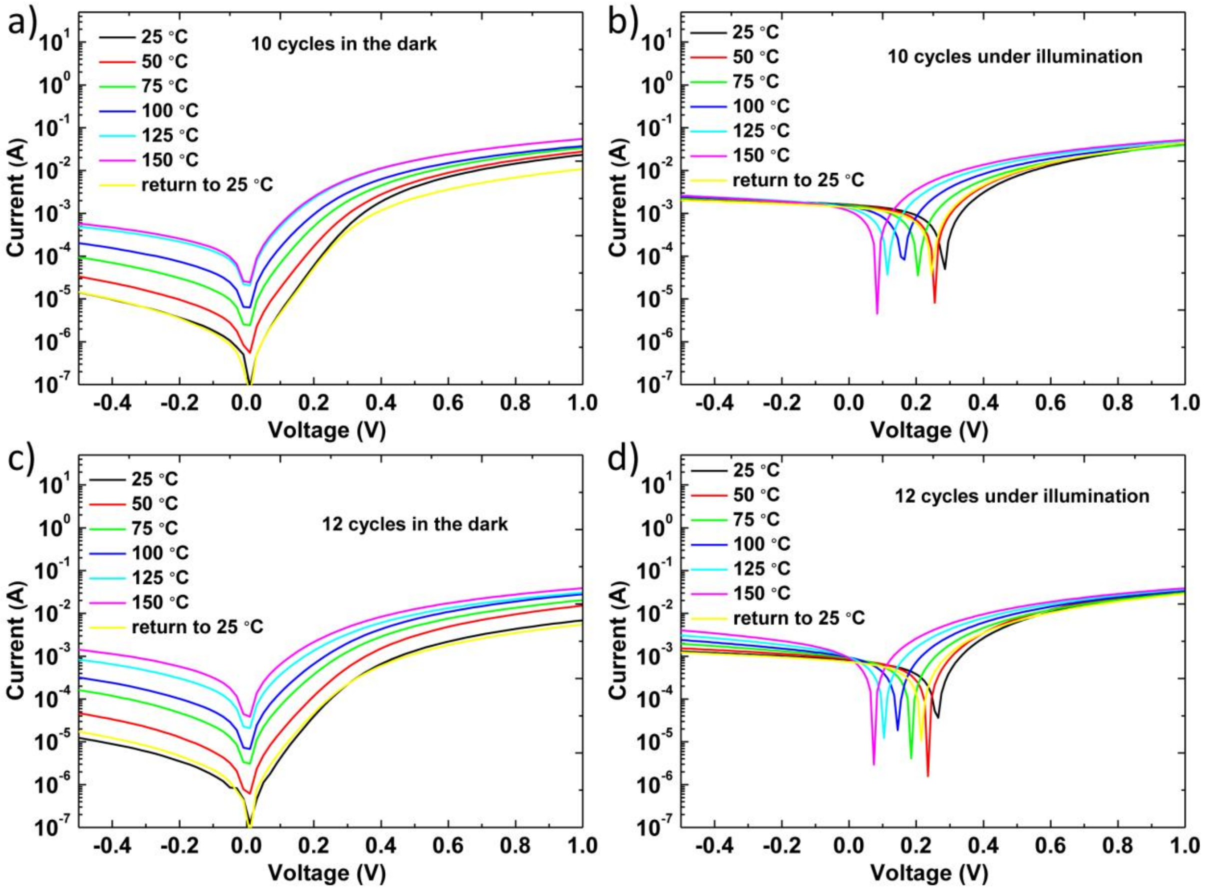

Figure S13. Current-voltage curves in the dark and under illumination of the SbSeI solar cells fabricated by (a, b) 10 cycles of spin-coating and thermal decomposition, and (c, d) 12 cycles of spin-coating and thermal decomposition.

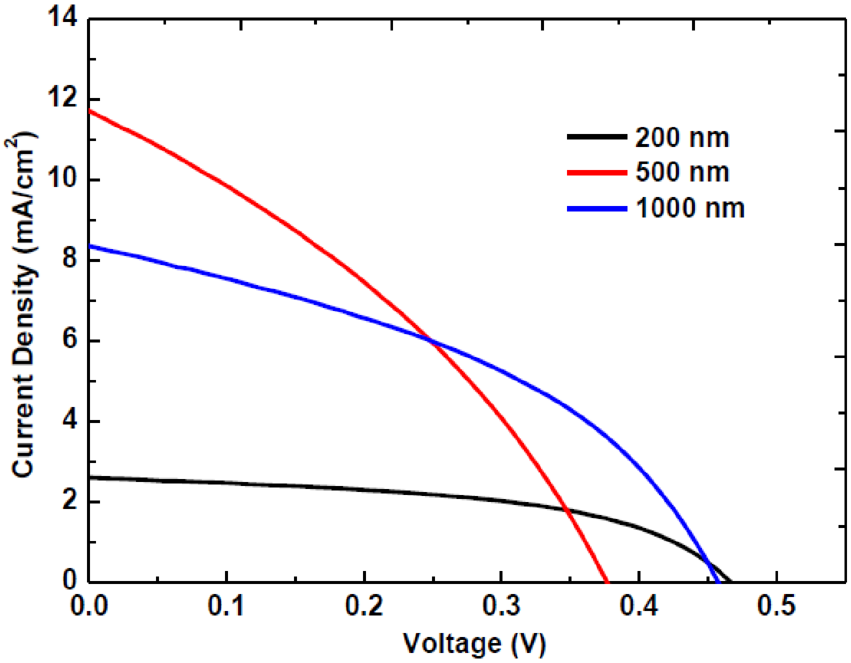

Figure S14. Current-voltage curves under illumination of the SbSeI solar cells with 200, 500 and 1000 nm mp-TiO_2.

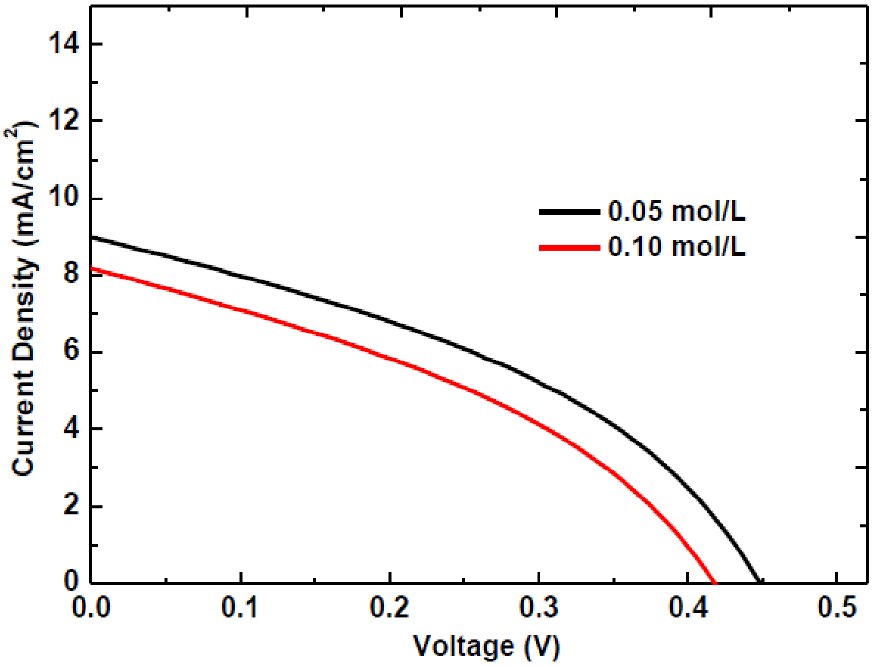

Figure S15. Current-voltage curves under illumination of the SbSeI solar cells fabricated using 0.05 and 0.10 mol/L Se-SSP solutions.

Table S1. The data extracted from Figure 3d. Here R_s and R_{REC} denote the series and recombination resistance, respectively, and CPE-T and CPE-P denote pseudocapacitances.

Deposition multi-cycle	Rs (Ω)	Rct (Ω)	CPE-T	CPE-P
10 cycles	168	2380	1.1134E-6	0.78
12 cycles	59.73	955.5	2.6204E-6	0.78

Table S2. PCEs of recent first solar cells.

	J_{SC} (mA/cm^2)	V_{OC} (V)	FF	PCE (%)	literature
**$CH_3NH_3PbI_3$ ($MAPbI_3$)**	11.0	0.61	0.57	3.81	J. Am. Chem. Soc. 2009, 131, 17, 6050–6051
**$CH_3NH_3PbBr_3$($MAPbBr_3$)**	5.57	0.96	0.59	3.13	J. Am. Chem. Soc. 2009, 131, 17, 6050–6051
**$Cs_3Bi_2I_9$**	2.15	0.85	0.60	1.09	Adv. Mater. 2015, 27, 6806–6813
**$MA_3Bi_2I_9$**	0.52	0.68	0.33	0.12	Adv. Mater. 2015, 27, 6806–6813
**Cs_2TiBr_6**	5.69	1.02	0.564	3.28	Joule 2018, 2, 558–570
**SbSI**	9.11	0.58	0.577	3.05	Adv. Energy Mater. 2018, 8, 1701901
**$Pb_2SbS_2I_3$**	8.79	0.61	0.582	3.12	ACS Energy Lett. 2018, 3, 2376–2382
**$Cs_2AgBiBr_6$**	3.93	0.98	0.63	2.43	J. Mater. Chem. A, 2017, 5, 19972–19981
**$FASnI_3$**	24.45	0.238	0.36	2.10	J. Mater. Chem. A, 2015, 3, 14996–15000
**$(CH_3NH_3)_3Sb_2I_9$ 0D**	1.0	0.896	0.55	0.49	ACS Energy Lett. 2016, 1, 309–314
**SbSeI**	14.77	0.47	0.587	4.10	This work
